# ZFP226 is a novel artificial transcription factor for selective activation of tumor suppressor KIBRA

**DOI:** 10.1038/s41598-018-22600-6

**Published:** 2018-03-09

**Authors:** Katrin Schelleckes, Boris Schmitz, Malte Lenders, Mirja Mewes, Stefan-Martin Brand, Eva Brand

**Affiliations:** 1 University Hospital Muenster, Internal Medicine D, Nephrology, Hypertension and Rheumatology, Albert-Schweitzer-Campus 1, 48149 Muenster, Germany; 2 University Hospital Muenster, Institute of Sports Medicine, Molecular Genetics of Cardiovascular Disease, Horstmarer Landweg 39, 48149 Muenster, Germany

## Abstract

KIBRA has been suggested as a key regulator of the hippo pathway, regulating organ size, cell contact inhibition as well as tissue regeneration and tumorigenesis. Recently, alterations of *KIBRA* expression caused by promotor methylation have been reported for several types of cancer. Our current study aimed to design an artificial transcription factor capable of re-activating expression of the tumor suppressor KIBRA and the hippo pathway. We engineered a new gene named ‘*ZFP226*′ encoding for a ~23 kDa fusion protein. ZFP226 belongs to the Cys2-His2 zinc finger type and recognizes a nine base-pair DNA sequence 5′-GGC-GGC-GGC-3′ in the *KIBRA* core promoter P1a. ZFP226 showed nuclear localization in human immortalized kidney epithelial cells and activated the *KIBRA* core promoter (p < 0.001) resulting in significantly increased KIBRA mRNA and protein levels (p < 0.001). Furthermore, ZFP226 led to activation of hippo signaling marked by elevated YAP and LATS phosphorylation. In Annexin V flow cytometry assays ZFP226 overexpression showed strong pro-apoptotic capacity on MCF-7 breast cancer cells (p < 0.01 early-, p < 0.001 late-apoptotic cells). We conclude that the artificial transcription factor ZFP226 can be used for target KIBRA and hippo pathway activation. This novel molecule may represent a molecular tool for the development of future applications in cancer treatment.

## Introduction

KIBRA (WWC1), a WW and C2 domain-containing protein has been identified as an upstream regulatory component of the hippo pathway (also known as Salvador/Warts/Hippo tumor suppressor network), which regulates cell number by modulating proliferation, apoptosis, and differentiation^1–4^. The hippo pathway is highly conserved in mammals and the ability of the WWC proteins to modulate hippo signal transduction and thus to inhibit cell proliferation has been proposed to be evolutionarily conserved from fly to men^5,6^. The hippo pathway negatively regulates the activity of two main downstream mediators: Yes-associated protein (YAP) and its family member the transcriptional co-activator with PDZ-binding motif (WWTR1/TAZ)^7–9^. Active YAP and TAZ translocate into the nucleus and promote proliferation by interaction with different transcription factors (TFs), including TEA domain family member (TEAD) 1–4^10^. Upon phosphorylation, YAP and TAZ are inactivated with subsequent cytoplasmic sequestration and eventual ubiquitination and degradation^10^. KIBRA acts as an upstream tumor suppressor protein that regulates hippo signaling in conjunction with neurofibromatosis-2 (NF2), preventing YAP and TAZ activation^1–3^.

In humans, impaired hippo signaling has been reported in a variety of different cancers, such as renal cell carcinoma^11^, hepatocellular carcinoma^12^ and breast cancer^13,14^, linking dysregulated hippo signaling to tumor initiation and progression^15–19^. Components of the hippo pathway are target of aberrant gene methylation and epigenetic silencing in humans^17^ as already reported for LATS1/2 (large tumor suppressor kinases 1 and 2; human Warts homologue)^20,21^, MST1/2 (serine/threonine protein kinase 4/3; human hippo homologue)^22,23^. *KIBRA* promoter methylation resulting in reduced KIBRA protein levels has been identified in chronic and acute lymphocytic leukemia^24,25^. Furthermore, alterations of *KIBRA* expression in clear cell renal cell carcinoma (ccRCC) have been analyzed in whole-genome expression profiling using Illumina BeadChip technology. The gene expression profiles of 101 ccRCC and adjacent tissue sample pairs of the K2 series suggested *KIBRA* downregulation in this series using locus-specific probes^26^. In this line, we observed that inactivated *KIBRA* expression depends on promoter methylation in ccRCC^27^.

Since aberrant epigenetic silencing of tumor suppressor genes (TSGs) plays a major role during tumorigenesis, regaining expression and effective normalization of function offers a unique opportunity for targeted therapies^28^. Novel and more precise medical strategies may involve artificial TFs for locus-specific modulation of gene expression and the reactivation of tumor suppressor function. This approach has been successfully used for the experimental treatment of breast, ovarian and cervical cancer cell lines with an artificial TF re-activating *EPB41L3* expression even when expression was silenced by promoter hypermethylation^29^. Here, we describe the activation of the tumor suppressor KIBRA using a novel artificial TF named ZFP226. ZFP226 induced KIBRA mRNA and protein, resulting in increased YAP phosphorylation and thus activation of hippo signaling. Furthermore, ZFP226 was capable of reducing the viability of MCF-7 breast cancer cells.

## Results

### Design of the artificial zinc finger ZFP226

**Figure 1:**
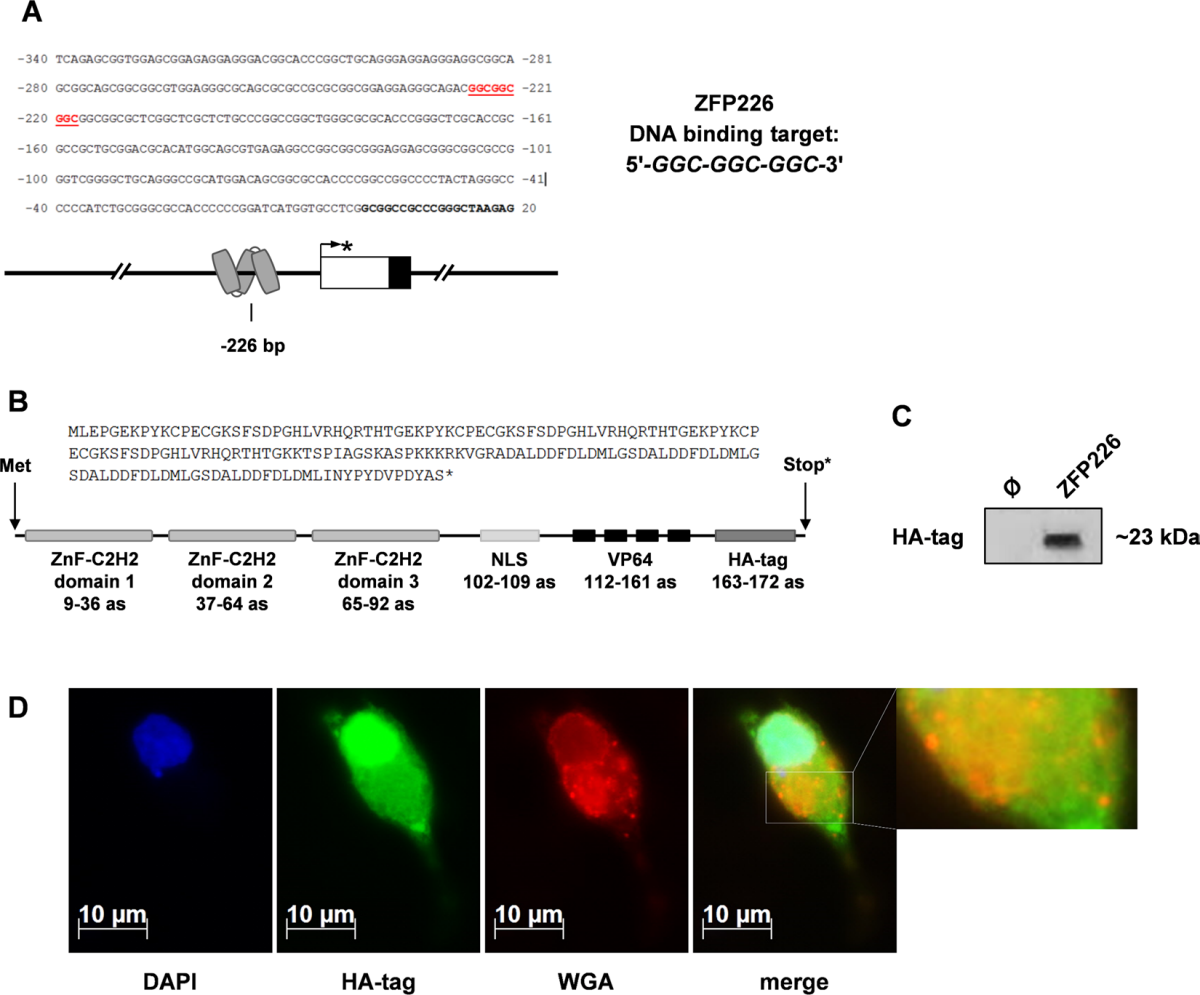
ZFP226 target sequence in the human *KIBRA* promoter, amino acid assembly and cellular localization. (**A**) The ZFP226 DNA target site. The artificial zinc finger ZFP226 was designed for specific binding of the 5′-GGC-GGC-GGC-3′ motif at position −226 bp within the human *KIBRA* promoter P1a. The *KIBRA* transcription start site TSS1 is marked by an asterisk. (**B**) Amino acid sequence (as) and functional domains of ZFP226. The ZFP226 peptide consists of 93 amino acids encoding for three zinc finger C2H2-domains, a nuclear localization signal (NLS, ‘PKKKRKV’), a VP64 activator domain of four VP16 motifs (‘DALDDFDLDML’) and an HA-tag (‘YPYDVPDYA’) resulting in a ~23 kDa fusion protein. (**C**) Expression of ZFP226. ZFP226 vector was transfected into IHKE cells followed by western blot detection using anti-HA antibody. The cropped blot is representative for experiments (n = 3) and the respective full-length blot is shown in Supplementary Fig. S2. (**D**) Cellular localization of ZFP226. A strong nuclear localization signal for ZFP226 in renal IHKE cells was detected by immunofluorescence using anti-HA antibody. DAPI was used for DNA staining, wheat germ agglutinin (WGA) was used for membrane staining.

KIBRA, a hippo pathway regulator, has been identified as a central TSG, which is frequently affected by epigenetic silencing in different types of cancer^1–3^. We recently reported that human *KIBRA* expression depends on a complex alternative CpG-rich promoter system^30^. Moreover, we observed that inactivated *KIBRA* expression depends on promoter methylation in ccRCC^27^. Based on this work, we generated an artificial zinc finger TF for *KIBRA* expression reactivation. The artificial three zinc finger ZFP226 belongs to the Cys2-His2 type and was constructed to target the DNA sequence 5′-GGC-GGC-GGC-3′ located within the human *KIBRA* core promoter P1a (Fig. 1A). This DNA target sequence was chosen for its location within a transcriptionally active region with high sensitivity for promoter methylation^30^. The ZFP226 sequence consists of 279 bp encoding for 93 amino acids. A nuclear localization signal (‘PKKKRKV’), a VP64 activator domain of four VP16 motifs (‘DALDDFDLDML’) and a HA-tag (‘YPYDVPDYA’) were linked to the ZFP226, resulting in a ~23 kDa (522 bp) fusion protein (Fig. 1B, Supplementary Fig. S1). The Cys2-His2 class of zinc finger proteins was chosen with respect to very specific characteristics. In brief, the structural stability of the Cys2-His2 zinc finger ββα-fold is based on hydrophobic interactions and chelation of a zinc ion by the Cys2-His2 residues^31^. Amino acid side chain contacts created by the α-helix of the domain are responsible for nucleic acid recognition^31^. The covalent linkage of multiple zinc finger domains subsequently allows for the recognition of extended asymmetric DNA sequences^31^ such as the *KIBRA* target 5′-GGC-GGC-GGC-3′ motif.

The artificial zinc finger ZFP226 was readily expressed in IHKE cells transfected with the generated expression plasmid (pZFP226) as detected by western blot targeting the HA-tag (Fig. 1C). Nuclear localization was confirmed by immunofluorescence using anti-HA antibody (Fig. 1D). WGA was used to stain cellular membranes (nuclear envelope, endoplasmic reticulum [ER], plasma membrane, vesicles). The merged image suggests ZFP226 localization also to membranous compartments such as the ER, endosomes and lysosomes within the cytosol (Fig. 1D, enlarged detail of merged image).

### Expression of ZFP226 leads to activation of the KIBRA core promoter

**Figure 2:**
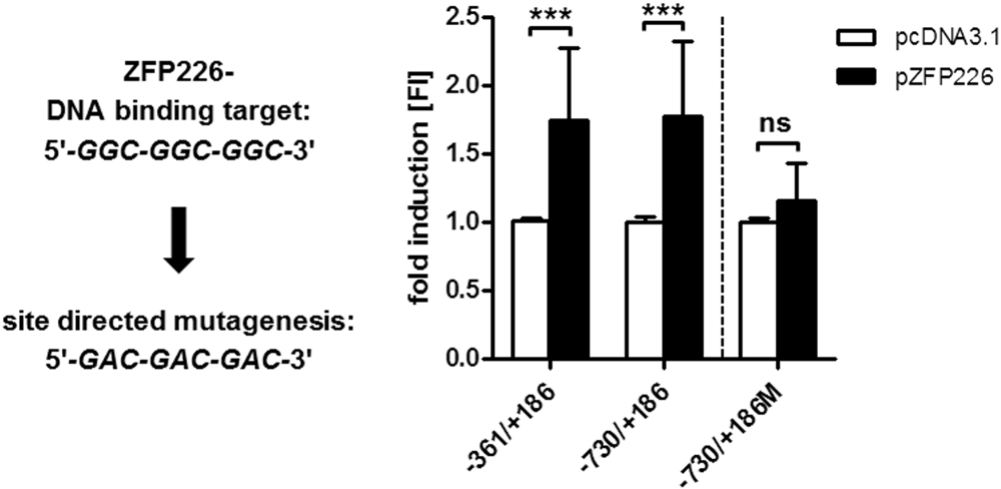
Target activation of *KIBRA* promoter P1a by ZFP226. Luciferase-based reporter experiments with ZFP226 co-expression resulted in a significant activation of *KIBRA* promoter P1a (constructs −316/+186 and −730/+186) compared to pcDNA3.1 mock-control. Site-directed mutagenesis (construct −730/+186 M) prevented the ZFP226 effect. Promoter activity of *KIBRA* reporter constructs was determined as relative light units and expressed as FI compared to mock-transfection. Transfections are representative for experiments (n = 4). ***p < 0.001; ns, not significant.

Luciferase-based cotransfection experiments using a *KIBRA* core promoter construct as reporter and the ZFP226 expression vector resulted in a ~1.7-fold increase of transcriptional activity for promoter construct −730/+186 compared to shuttle vector control (p < 0.001, Fig. 2) in IHKE cells. Consistently, ZFP226 binding-site mutation prevented the activating effect of ZFP226 (p = 0.0731, Fig. 2). This result suggests that ZFP226 is able to drive *KIBRA* gene expression by target promoter activation.

### ZFP226 induces the upregulation of endogenous KIBRA

**Figure 3:**
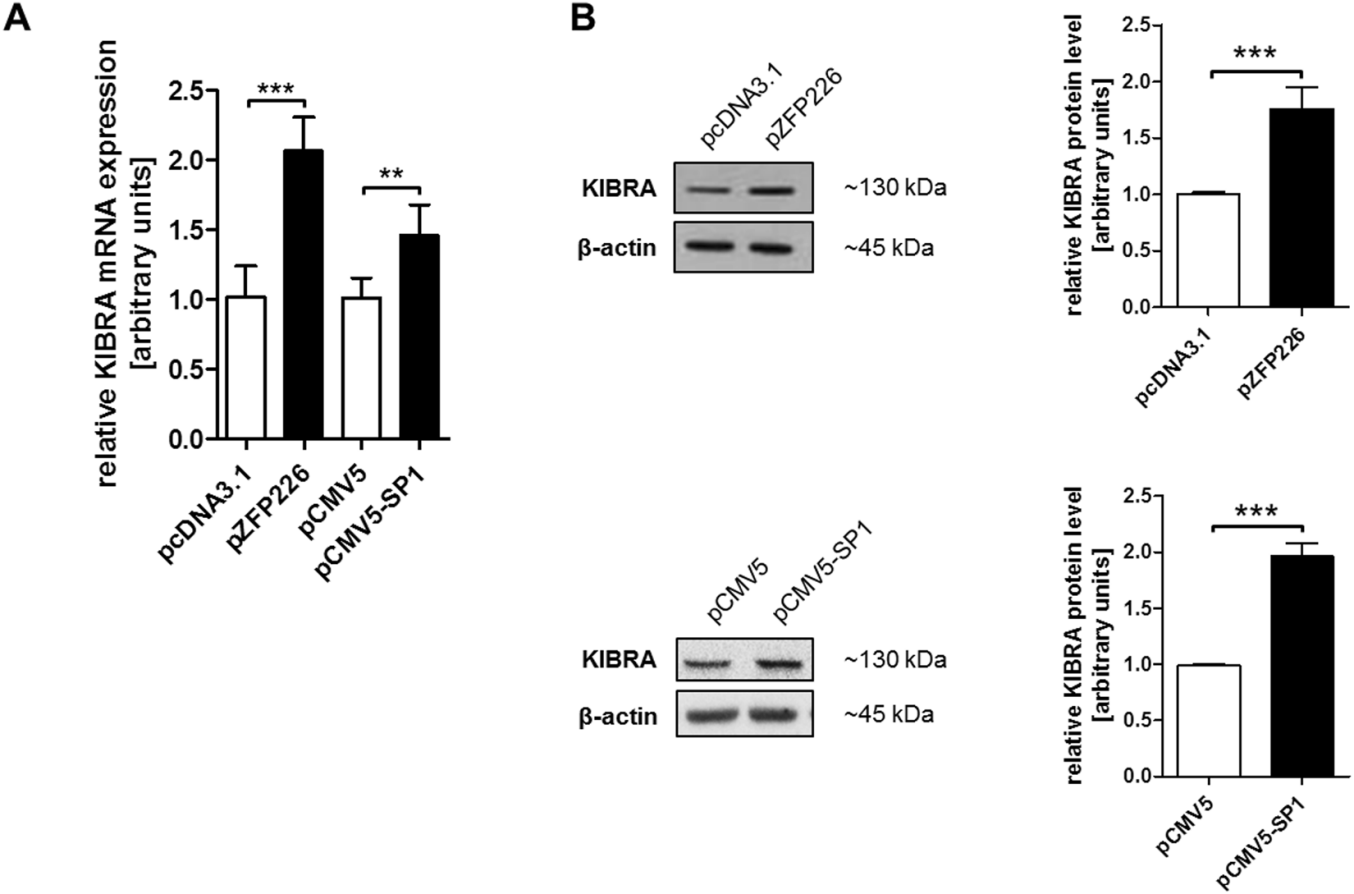
ZFP226 overexpression activates KIBRA mRNA and protein expression. *KIBRA* expression levels increased significantly 48 hrs after ZFP226 transfection detected by (**A**) real-time PCR and (**B**) western blot. The activating effect of ZFP226 was compared to SP1 as positive control. The cropped blots are representative for experiments (n = 4), densitometry is based on independent experiments as indicated. Beta actin was used as loading control. The respective full-length blots are shown in Supplementary Fig. S3. **p < 0.01, ***p < 0.001.

To study the cellular consequences induced by the generated artificial zinc finger ZFP226, we analyzed KIBRA mRNA and protein expression levels in transfected IHKE cells. Real-time PCR analysis revealed significantly increased KIBRA mRNA expression levels 48 hrs after ZFP226 transfection compared to shuttle vector control (all p < 0.001, Fig. 3A). Of note, our previous analyses revealed that ubiquitous TF SP1 is a strong activator of KIBRA expression on mRNA and protein level^27^. Therefore, SP1 overexpression served as positive control in real-time PCR and western blot experiments. KIBRA protein was significantly increased by SP1 and ZFP226 compared to shuttle vector control (p < 0.001, Fig. 3B). These results suggest that the artificial TF ZFP226 exhibits comparable biological activity to drive KIBRA expression as TF SP1.

### ZFP226 activates YAP and LATS1 phosphorylation

**Figure 4:**
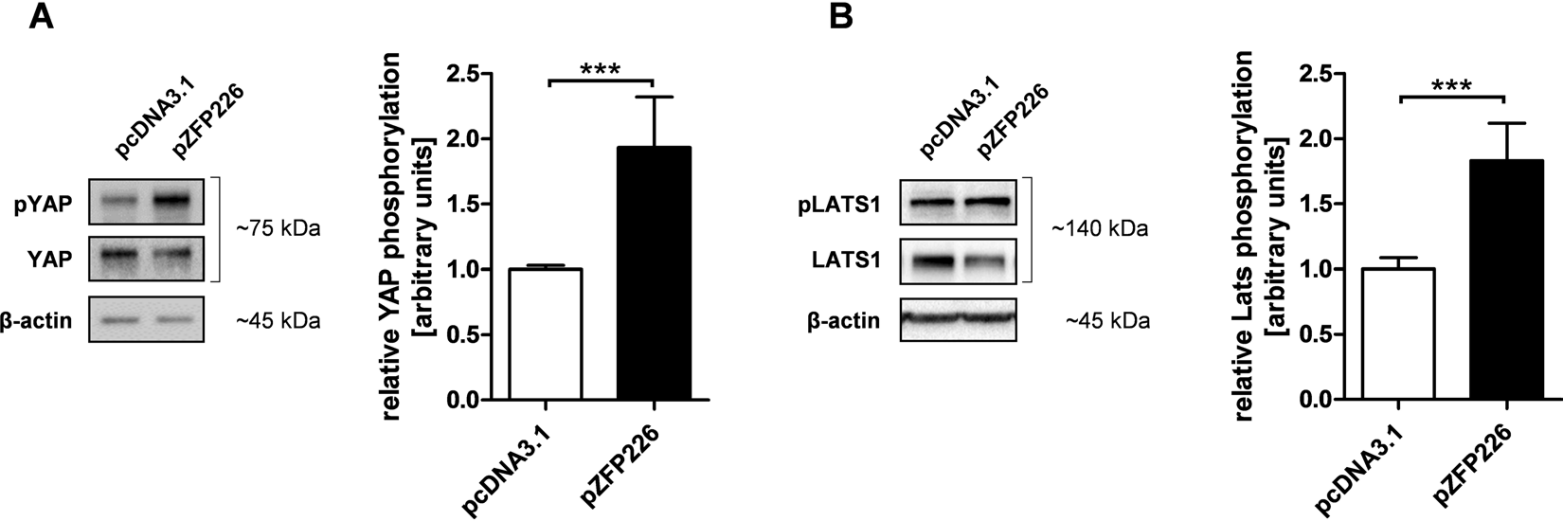
ZFP226 overexpression enhances YAP and LATS1 phosphorylation. After ZFP226 transfection phosphorylation of (**A**) YAP (pYAP) and (**B**) LATS1 (pLATS1) increased significantly detected by western blot. Relative YAP and LATS1 phosphorylation was calculated using beta-actin as loading control and total YAP/LATS1 as reference. The cropped blots are representative for experiments (YAP, n = 5; LATS1, n = 4), densitometry is based on independent experiments as indicated. The respective full-length blots are shown in Supplementary Figs S5, S6. ***p < 0.001.

Since active hippo signaling leads to LATS1 phosphorylation und subsequent YAP inactivation with cytosolic sequestration and eventual degradation, relative LATS1 and YAP phosphorylation (pLATS1, pYAP) are important indicators of hippo pathway activity. We were able to show that relative levels of pLATS1 and most importantly pYAP were significantly increased 48 hrs after ZFP226 transfection in IHKE cells (both p < 0.001, Fig. 4A,B), suggesting an activation of hippo signaling by elevated *KIBRA* expression. Of Note, YAP expression was unaffected by pZFP226 transfection.

### ZFP226 induces apoptosis of breast cancer cells

In cancer, defects in different apoptotic pathways can disturb the balance between cell proliferation and apoptosis, and thus allow the survival of cells with genetic abnormalities^32^. The hippo pathway has been shown to regulate cell number by modulating proliferation, differentiation, and apoptosis^1–4^. Furthermore, impaired hippo signaling has been reported in a variety of different cancers, including breast cancer^13,14^, linking dysregulated hippo signaling to tumor initiation and progression^15–19^. Therefore, we hypothesized that ZFP226 may induce apoptosis in human MCF-7 breast adenocarcinoma cells by activating hippo signaling.

**Figure 5:**
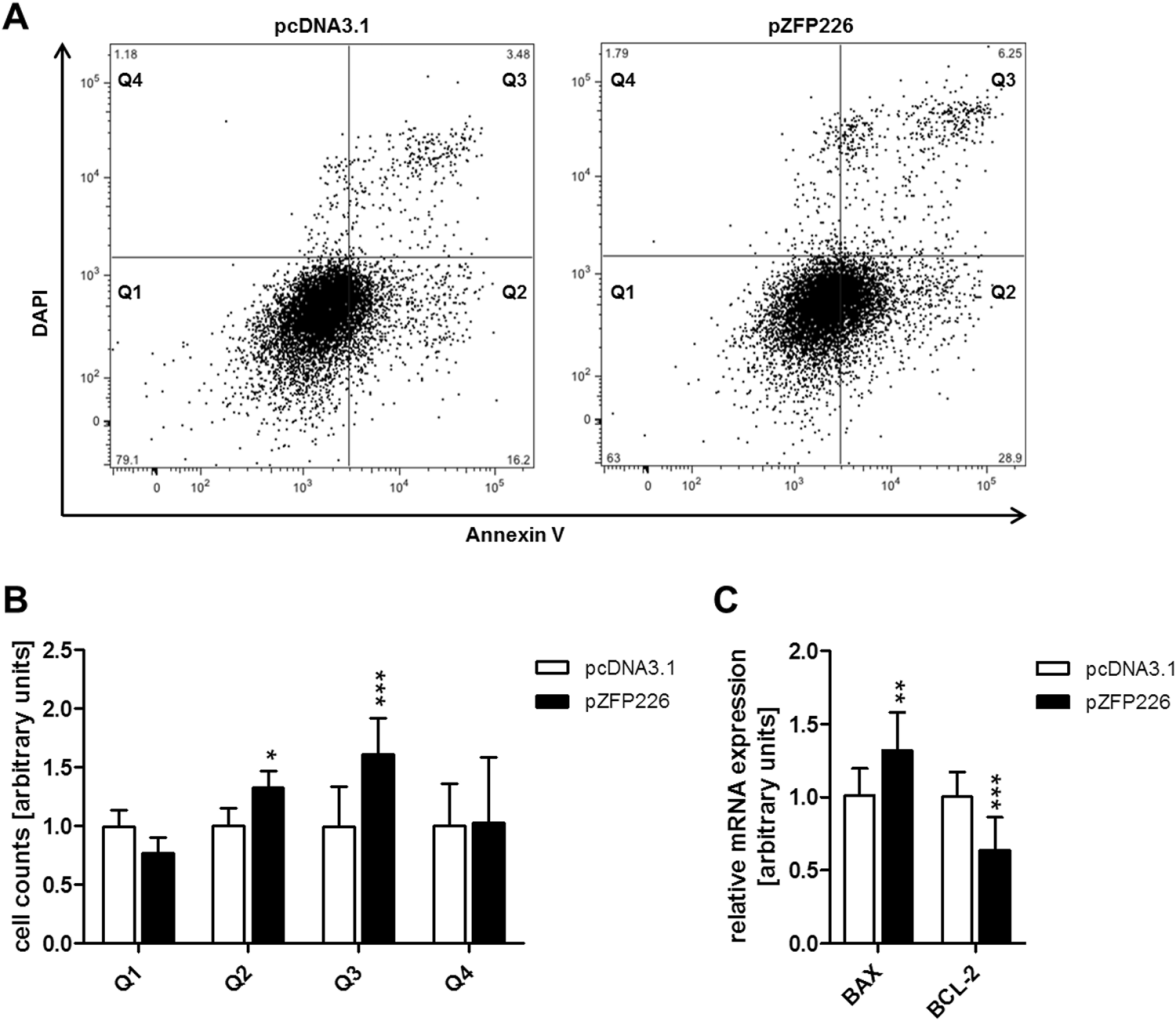
ZFP226 induced apoptosis in MCF-7 cells. Apoptosis of pZFP226-transfected MCF-7 cells was measured by Annexin-V/DAPI staining using flow cytometry. (**A**) Representative flow cytometry graphs showing the distribution of cells. Live cells (Q1 Annexin V−/DAPI−), early-apoptotic cells (Q2 Annexin V+/DAPI−), late-apoptotic/necrotic cells (Q3 Annexin V+/DAPI+) and cell debris (Q4 Annexin V−/DAPI+). (**B**) The number of apoptotic cells within quadrant Q2 and Q3 was significantly increased 48 hrs after ZFP226 transfection. (**C**) A significant increase for pro-apoptotic BAX and a significant decrease for anti-apoptotic BCL-2 was detected by real-time PCR 48 hrs after ZFP226 transfection. Flow cytometry and real-time PCR analysis are representative for experiments (n = 4). *p < 0.05, **p < 0.01, ***p < 0.001.

Cells were transfected with pZFP226, incubated for 48 hrs and analyzed by flow cytometry (Fig. 5A,B, Supplementary Fig. S4). The number of apoptotic cells within quadrant Q2 (early-apoptotic cells) and quadrant Q3 (late-apoptotic/necrotic cells) was significantly increased after ZFP226 transfection in MCF-7 cells compared to shuttle vector control (p_Q2_ < 0.05, p_Q3_ < 0.01; Fig. 5B). Additionally, real-time PCR analysis of apoptotic markers revealed significantly increased pro-apoptotic BAX as well as decreased anti-apoptotic BCL-2 expression after ZFP226 transfection compared to shuttle vector control (p_BAX_ < 0.01, p_BCL-2_ < 0.001; Fig. 5C).

## Discussion

In the current study we report the design, construction and functional characterization of the novel artificial TF ZFP226. ZFP226 was capable to (1) activate the *KIBRA* core promoter, a tumor suppressor and upstream regulator of the hippo pathway, resulting in (2) significantly increased KIBRA mRNA as well as protein levels, (3) activation of hippo signaling marked by elevated LATS1 and YAP phosphorylation and (4) reduced viability of breast cancer cells.

In humans, impaired hippo signaling has been reported for several types of cancer^16,33^ such as renal cell carcinoma^11^, hepatocellular carcinoma^12^ and breast cancer^13,14^. In line with this observation, components of the hippo pathway are target of aberrant gene methylation and epigenetic silencing also in humans^17^ as already reported for *LATS1/2*^20,21^, *MST1/2*^22,23^ and *KIBRA*^24,25^. We recently reported that human *KIBRA* expression depends on a complex alternative CpG-rich promoter system^30^ with inactivated *KIBRA* expression induced by promoter methylation in ccRCC^27^. Based on this work, we generated the artificial zinc finger ZFP226 to activate the expression of tumor suppressor KIBRA and (re-)activation of the hippo pathway. Artificial zinc finger TFs have been suggested as a powerful molecular tools to modulate target gene expression in cells and organisms. These TFs are designed to specifically recognize target sites within the promoter region of interest and effectively up- or downregulate expression of their target genes not only *in vitro*, but also *in vivo*^34–38^. This approach has been successfully used for the experimental treatment of breast, ovarian and cervical cancer cell lines with an artificial TF re-activating *EPB41L3* expression even when expression was silenced by promoter hypermethylation^28^. Furthermore, Cori *et al*.^35^ engineered an artificial zinc finger-based TF (“Jazz”) for upregulation of the human and mouse *utrophin* expression^35,36^. Mattei *et al*.^37^ generated transgenic mice that specifically overexpressed “Jazz” in skeletal muscle. Subsequently, crossing the “Jazz” transgenic mice with the Duchenne muscular dystrophy mouse model resulted in a strong amelioration of the dystrophic phenotype^37,38^. These examples provide evidence for the promising therapeutic approach based on artificial TFs.

ZFP226 belongs to the Cys2-His2 zinc finger type and recognizes a nine base pair DNA sequence 5′-GGC-GGC-GGC-3′ in the *KIBRA* core promoter P1a, which is well-characterized^30^. Artificial TFs are designed to target a single promoter but may have multiple DNA targets by chance. Assuming random base distribution, a nine base pair DNA sequence such as 5′-GGC-GGC-GGC-3′, is present in the human genome about 1.3 × 10^4^ times^35^. However, only a small portion of these sequences is accessible to DNA-binding proteins with an active role in gene expression regulation. Even if we were able to demonstrate the ability of ZFP226 to activate the *KIBRA* core promoter resulting in significantly increased KIBRA mRNA and protein levels, further improvement of ZFP226 specificity by target sequence extension is mandatory.

The hippo pathway negatively regulates the activity of two main downstream mediators, YAP and its family member TAZ^7–9^. YAP and TAZ are inactivated by phosphorylation through LATS kinases with subsequent inhibition of proliferation^10^. Therefore, YAP protein level and YAP phosphorylation are important indicators of hippo pathway activity. Of note, in datasets of breast cancer patients, elevated expression of gene signatures for YAP/TAZ activity correlated with high histological grade, enrichment of stem cell signatures, metastasis development and progression, as well as poor outcome^39–41^. To this respect, enriched TAZ nuclear staining has been detected in high-grade breast cancer^40,42^ and is associated poor clinical outcome^42,43^. Furthermore, in primary breast cancer specimens reduced *KIBRA* expression has been correlated with the claudin-low subtype, an aggressive sub-group with epithelial-to-mesenchymal transition features and a poor prognosis^44^. In our experiments, we detected a significant activation of hippo signaling in ZFP226-transfected cells marked by elevated LATS1 and YAP phosphorylation. Of note, LATS1 protein levels tended to be decreased in individual ZFP226 experiments suggesting a potential feedback mechanism to restore hippo pathway homeostasis^5^. However, this trend was not statistically significant over all analyzed experiments. Most importantly, we found that ZFP226 was able to induce apoptosis by activating hippo signaling in human breast adenocarcinoma cells as we observed a significantly increased number of early- and late-apoptotic cells after transfection with ZPF226. These findings were supported by significantly elevated expression of pro-apoptotic BAX and decreased expression of anti-apoptotic BCL-2 within ZFP226-transfected cells.

## Conclusion

ZFP226 is a novel artificial TF, which was capable to activate the *KIBRA* core promoter, to significantly increase KIBRA mRNA as well as protein levels, thereby activating hippo signaling marked by elevated LATS1 and YAP phosphorylation. ZFP226 reduced the viability of breast cancer cells *in vitro*. This novel molecule may represent a molecular tool for the development of future applications in cancer treatment and needs further investigation.

## Methods

### Construction of the ZFP226 transcription factor

The artificial zinc finger protein ZFP226 was constructed using *ZF tools 3.0* as described previously^45^. ZFP226 was engineered to drive transcription from the 9 base pair DNA sequence 5′-GGC-GGC-GGC-3′ located to *KIBRA* promoter P1a^30^. The resulting *ZFP226* sequence consists of 279 bp encoding for 93 amino acids. A nuclear localization signal (‘PKKKRKV’), a VP64 activator domain of four VP16 motifs (‘DALDDFDLDML’) and an HA-tag (‘YPYDVPDYA’) were fused C-terminal to the ZFP226, resulting in a ~23 kDa (522 bp) protein. Off-target binding was controlled using data from ELISA specificity graphs (implemented in ZF tools 3.0). The final sequence was synthesized and ligated into the pEX-A2 vector by Eurofins Genomics. ZFP226 cDNA was then hydrolyzed using *Xho*I and *Kpn*I and subcloned into the pCMV-driven eukaryotic expression vector pcDNA3.1+ (Thermo Scientific; Supplementary Fig. S6). Sequence accuracy and identity was controlled by direct sequencing of both DNA strands.

### Cell culture, transfection and reporter gene assay

Immortalized human kidney epithelial cells (IHKE) were maintained in DMEM/Ham’s-F12 (Thermo Scientific) enriched with 5% fetal bovine serum (FBS; Sigma-Aldrich), 100 units/ml penicillin, 100 ng/ml streptomycin, 2 mmol/ml L-glutamine, 10 ml/l insulintransferrin-sodium selenite media supplement, 1.25 g/l NaHCO_3_, 55 mg/l sodium pyruvate, 10 µg/l human epidermal growth factor (all Thermo Scientific) and 15 mmol/l N2hydroxyethylpiperazineN2ethanesulfonic acid (HEPES; Merck)^46–48^. MCF-7 human breast adenocarcinoma cells were maintained in DMEM enriched with 10% FBS, 100 units/ml penicillin, 100 ng/ml streptomycin, 2 mmol/ml L-glutamine and 55 mg/l sodium pyruvate. IHKE cells were transfected using either jetPEI (Polyplus transfection; 1 µg DNA) or the Neon Transfection System (Thermo Scientific; 1100 V, 1 pulse, 30 ms; 3 µg DNA) according to the manufacturer’s instruction for 48 h. Luciferase-based reporter gene assays were performed as described^49^. In brief, ZFP226 or SP1 expression vector or the appropriate shuttle vector control and *KIBRA* reporter gene plasmids for promoter P1a (−361/+186 and −730/+186)^30^ were transfected in a 1:1 ratio. The *KIBRA* promoter construct −730/+186 was mutated by sitedirected mutagenesis (oligonucleotide sequences are given in Supplementary Table T1). Luciferase activities were measured using the luciferase assay kit (Promega) and a Sirius luminometer (Berthold detection systems). All vectors were sequenced to ensure sequence accuracy and identity. Transfection experiments were repeated three times.

### Western blot

For crude protein extracts, cells were lysed in RIPA buffer containing 1% NP40 and 0.1% SDS (Carl Roth) supplemented with ‘Complete’ and ‘PhosStop’ protease/phosphatase inhibitor cocktail (Sigma Aldrich) as described^50^. Immunodetection of cellular extracts was performed using an anti-KIBRA (Santa Cruz Biotechnology; 1:500), anti-SP1 (Merck; 1:1000), anti-YAP (Santa Cruz Biotechnology; 1:1000), anti-pYAP (Ser127; Cell Signaling; 1:1000), anti-LATS1 (Merck; 1:1000), anti-pLATS1 (Thr1079; Cell Signaling; 1:500) and anti-rabbit secondary antibody (Santa Cruz Biotechnology; 1:20000 or Merck; 1:10000). Sample loading was controlled by β-actin detection (Cell Signaling; 1:5000) and anti-rabbit secondary antibody (Santa Cruz Biotechnology; 1:10000 or Merck; 1:20000). ZFP226 detection was conducted using anti-HA antibody (Cell Signaling; 1:1000) and anti-mouse secondary antibody (Santa Cruz Biotechnology; 1:20000). Western blots were repeated at least three times and band intensities were quantified using ImageJ^51^.

### Immunofluorescence

A total of 2 × 10^5^ IHKE cells per 24-well were seeded on coverslips, transfected with pZFP226 and incubated for 48 hrs. Cells were washed with PBS twice, fixed in 4% paraformaldehyde (Sigma Aldrich) and permeabilized using 0.2% Triton X-100 (Sigma Aldrich) at 4 °C for 30 min. Cells were washed 3 times with PBS and incubated overnight at 4 °C with blocking solution (5% [w/v] BSA in PBS, 0.2% [v/v] Triton X-100). Subsequently, cells were incubated for 2.5 hrs at RT with primary antibodies against HA-tag (Cell signaling; 1:50). Alexa Fluor 488 anti-rabbit (Thermo Scientific; 1∶1000) secondary antibody was used for detection. DAPI (0.25 mg/ml; Roche) was used for DNA staining and wheat germ agglutinin (WGA) conjugated to Alexa Fluor 594 (Thermo Scientific) was used to stain membranes. Cells were imaged on an AxioObserver Z1 microscope (Carl Zeiss) using a 40× oil immersion objective, with identical exposure times.

### Real-time PCR

Total RNA was extracted using the NucleoSpin RNA Kit (Macherey-Nagel). First strand cDNA synthesis was performed using MuLV Reverse Transcriptase (Thermo Scientific) and 1 µg of total RNA. cDNA was amplified in a 384-well format (standard real-time PCR conditions) in duplicates using Power SYBR Green (Thermo Scientific) on an Applied Biosystems 7500 Fast real-time PCR system. Relative quantification was calculated using the 2^−ΔΔCt^ method and S18 as endogenous control. The absence of non-specific amplification products was confirmed by agarose gel electrophoresis and generation of melting curves using the Applied Biosystems software. Oligonucleotides had an amplification efficiency of ≥90%. BAX and BCL-2 were used as standard markers for apoptosis as described^52^ (oligonucleotide sequences are given in Supplementary Table S1).

### Annexin V apoptosis assay

For Annexin V apoptosis assays, MCF-7 cells were transfected with pZFP226 or pcDNA3.1 as shuttle vector control using the Neon Transfection System (Thermo Scientific) according to the manufacturer’s protocol. In brief, a total of 5 × 10^6^ cells/ml was transfected with 1 μg of plasmid DNA using the following configuration: 1100 V, 2 pulse, 30 ms. Then, 5 × 10^4^ cells/ml were seeded on a 6-well plate and incubated for 48 hrs. Cells were harvested using trypsin, spun down and washed with PBS twice. Cells were labelled for Annexin V as described^53^ using APC (allophycocyanin)-conjugated Annexin V (1:20; BD biosciences). Cells were DAPI -stained directly before measurement. Cells were sorted using BD FACSCanto II (BD biosciences). Diva software v6.1.3 was used and data analysis was performed with FlowJo software v9.5.1.

Flow cytometry data is presented in four quadrants (Q). Live cells; (Q1 Annexin V−/DAPI−), early-apoptotic cells; (Q2 Annexin V+/DAPI−), late-apoptotic/necrotic cells; (Q3 Annexin V+/DAPI+) and cell debris (Q4 Annexin V−/DAPI+).

### Statistical analysis

Data are given as mean ± SD p-values were calculated by unpaired, two-tailed Student’s t-test or one-way ANOVA where appropriate. p-values < 0.05 were considered significant.

### Data availability

The datasets generated and/or analysed during the current study are available from the corresponding author upon reasonable request.

## Electronic supplementary material

Supplementary Material
