# Supplementary material for: ZFP226 is a novel artificial transcription factor for selective activation of tumor suppressor KIBRA
Source: Sci Rep. 2018 Mar 9;8:4230. doi: 10.1038/s41598-018-22600-6 (PMC5844865; doi:10.1038/s41598-018-22600-6)
Supplement: Supplementary file 1 — Supplementary Material [file 41598_2018_22600_MOESM1_ESM.doc]

**ZFP226 is a novel artificial transcription factor for selective activation of tumor suppressor KIBRA**

Katrin Schelleckes^1*^; Boris Schmitz^2*^; Malte Lenders^1^; Mirja Mewes^1^; Stefan-Martin Brand^2^ and Eva Brand^1^

*these authors contributed equally to this work

^1^University Hospital Muenster, Internal Medicine D, Nephrology, Hypertension and Rheumatology, Albert-Schweitzer-Campus 1, 48149 Muenster, Germany

^2^University Hospital Muenster, Institute of Sports Medicine, Molecular Genetics of Cardiovascular Disease, Horstmarer Landweg 39, 48149 Muenster, Germany

**SUPPLEMENTARY MATERIAL**

**Supplementary table**

**Supplementary table T1**: Sequences and positions of oligonucleotides used in this study.

| **Oligonucleotide** | **Sequence 5'- 3'** | **Position; ref. acc. #** |
| --- | --- | --- |
| **Oligonucleotide sequences for generation of serial promoter deletion constructs** | | |
| KIBRA_ss1 | GACTCCTGATTCTACACCAC | -730; AC026689 |
| KIBRA_ss2 | GAAGTCACAAACCCGGCGA | -361; AC026689 |
| KIBRA_as1 | GGTGCGGTTCGTGTGGTC | +186 ; AC026689 |
| **Oligonucleotide sequences used for real-time PCR** | | |
| KIBRA_ss3 | CTTCGACGGCAAGGTCTAC | exon 1/2; NM_015238 |
| KIBRA_as2 | GCGGTTTGGTGTACCTGTC | exon 1/2; NM_015238 |
| BAX_ss | CCTGTGCACCAAGGTGCCGGAACT | exon 2; NM_001291430 |
| BAX_as | CCACCCTGGTCTTGGATCCAGCCC | exon 2; NM_001291430 |
| BCL-2_ss | CTGCACCTGACGCCCTTCACC | exon 1; NM_000633 |
| BCL-2_as | CACATGACCCCACCGAACTCAAAGA | exon 1; NM_000633 |
| **Oligonucleotide sequences for site-directed mutagenesis** | | |
| KIBRA_ss4 | GAGGGCAGACGGAGGCGACGACGACGCTCGGCTCG | ZFP226 binding site |
| KIBRA_as3 | CGAGCCGAGCGTCGTCGTCGCCTCCGTCTGCCCTC | ZFP226 binding site |

**Supplementary figures**

**Supplementary figure S1**

**
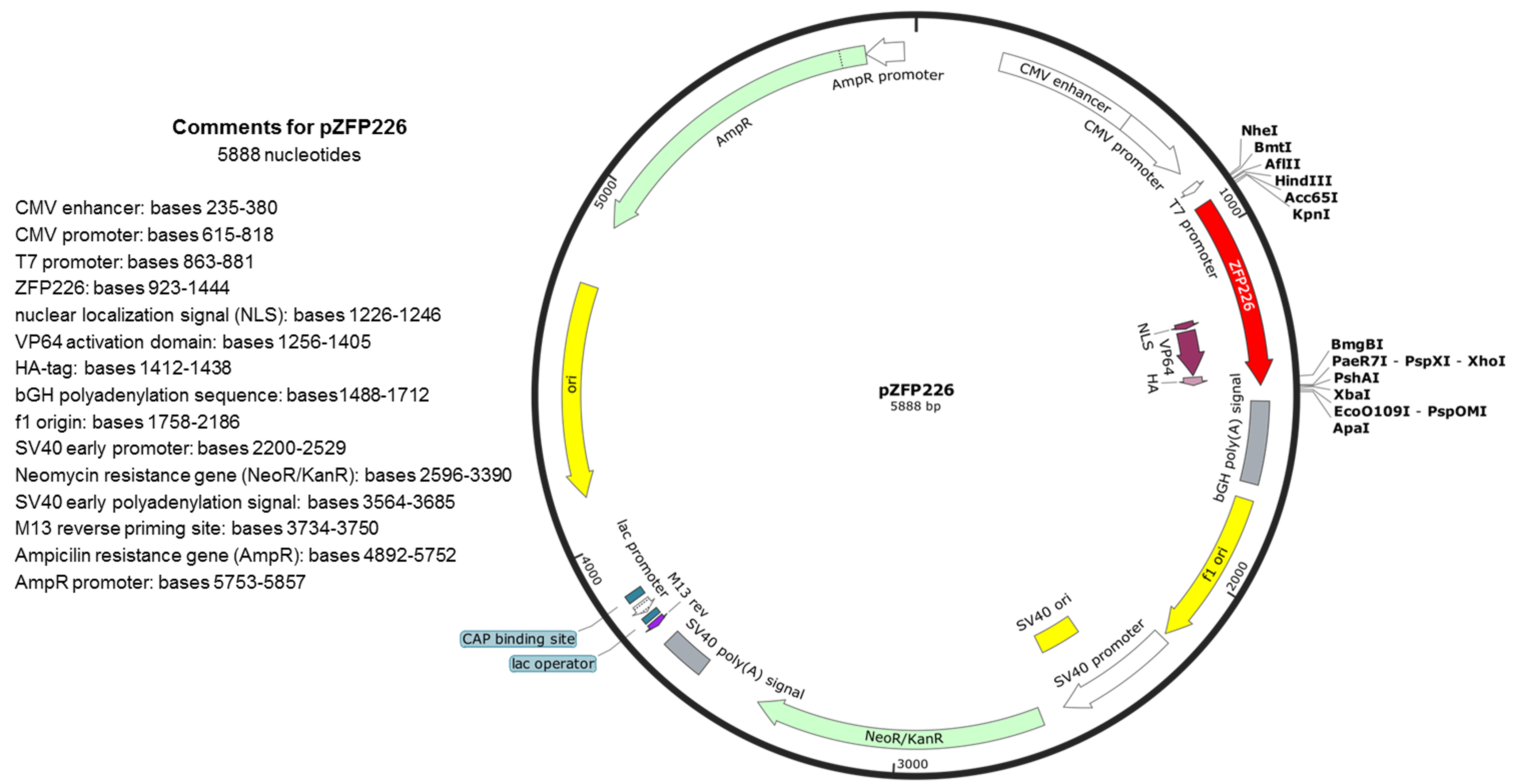
**

**Features of the pZFP226 vector.** ZFP226 cDNA (red arrow).

**Supplementary figure S2**

**
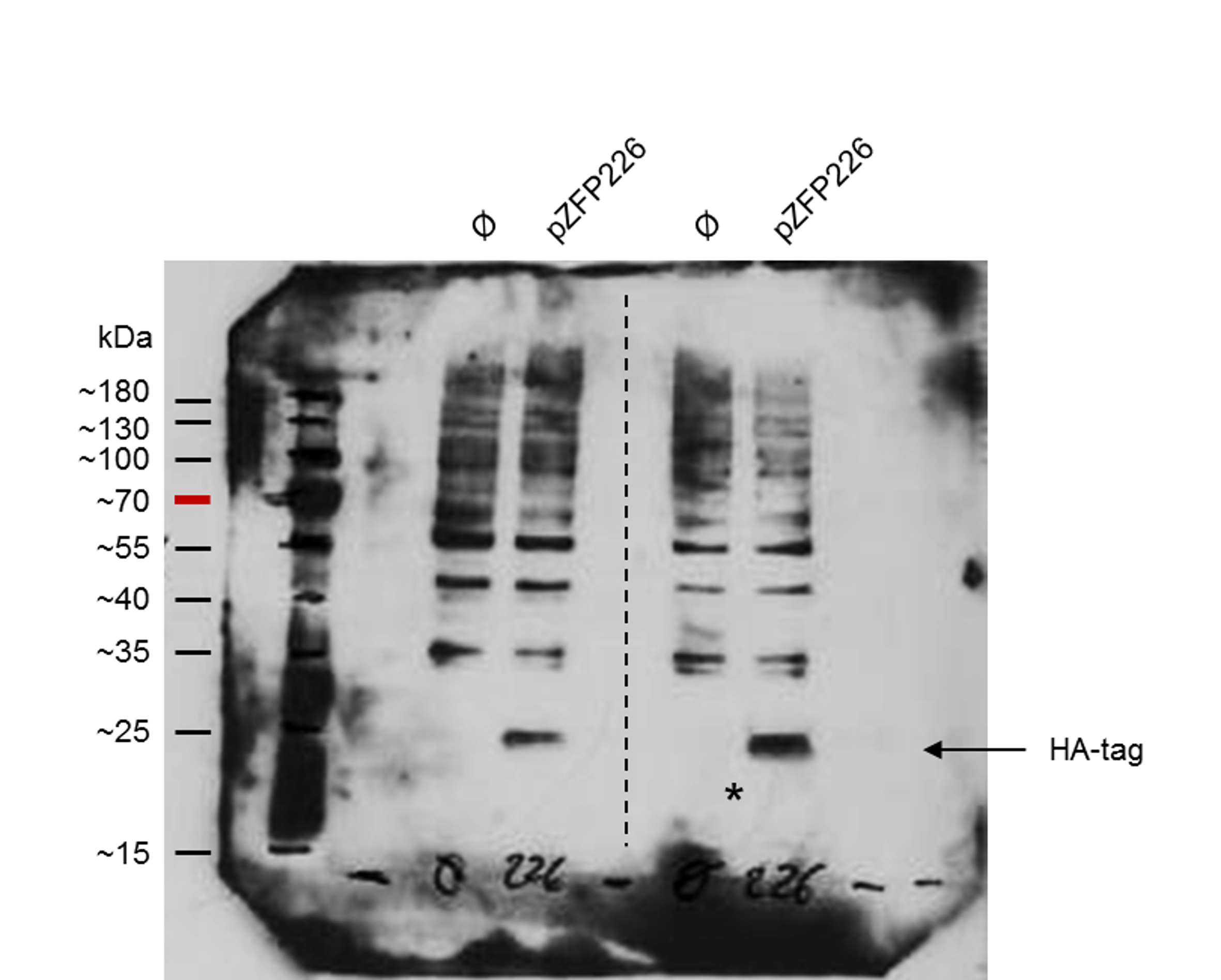
**

**Expression of ZFP226.** Uncropped image of the blot used in Fig. 1C. ZFP226 vector was transfected into IHKE cells followed by western blot detection using anti-HA antibody (Cell Signaling; 1:1000) and anti-mouse secondary antibody (Santa Cruz Biotechnology; 1:20000). Asterisk marks the cropped image used in Fig 1C.

**Supplementary figure S3**

**
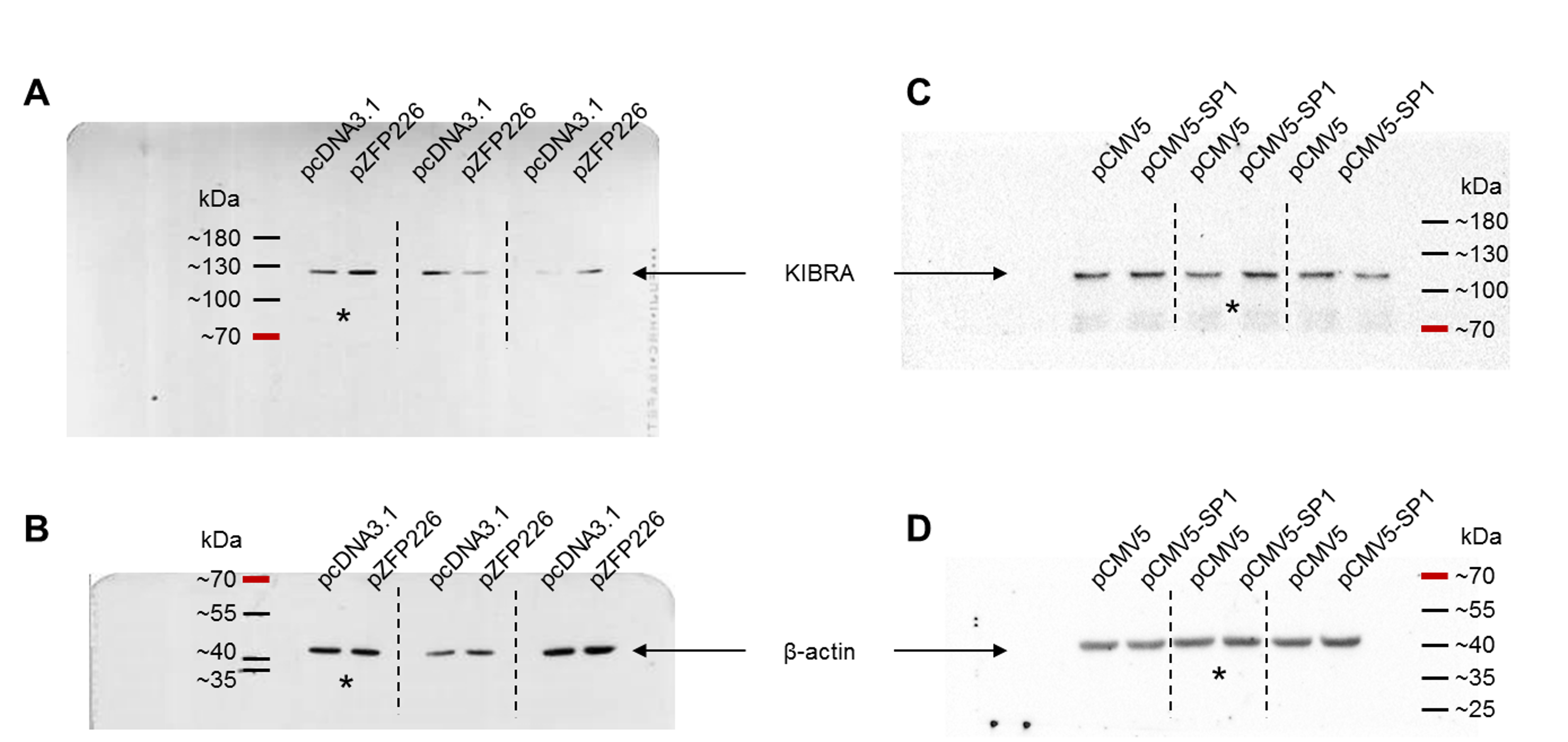
**

**ZFP226 overexpression activates KIBRA expression.** Uncropped images of the blots used in Fig. 3B. **(A-B)** ZFP226 or **(C-D)** SP1 vector was transfected into IHKE cells followed by western blot detection using an anti-KIBRA (Santa Cruz Biotechnology; 1:500), anti-SP1 (Millipore; 1:1000) and anti-rabbit secondary antibody (Santa Cruz Biotechnology; 1:20000). pcDNA3.1 and pCMV5 served as shuttle vector control. Sample loading was controlled by β-actin detection (Cell Signaling; 1:5000) and anti-rabbit secondary antibody (Santa Cruz Biotechnology; 1:10000). Asterisk marks the cropped image used in Fig. 3B.

**Supplementary figure S4**

**
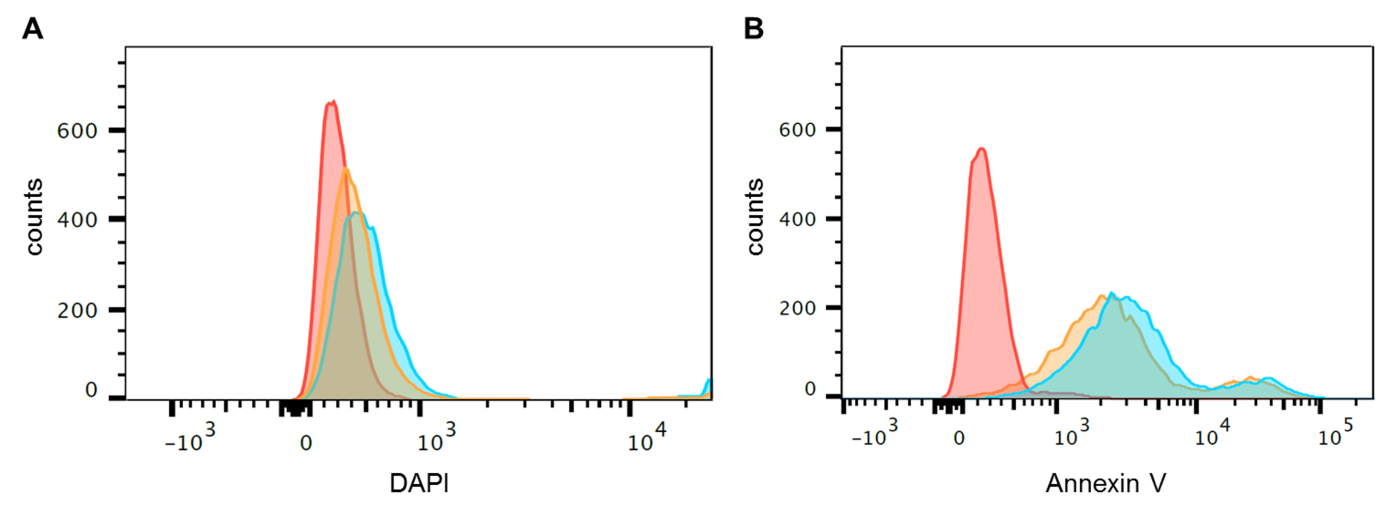
**

**Representative flow cytometry histograms.** The histograms show cell distributions of **(A)** DAPI- and **(B)** Annexin V-stained pcDNA3.1- (yellow) and pZFP226-transfected (blue) MCF-7 cells compared to unstained controls (red).

**Supplementary figure S5**

**
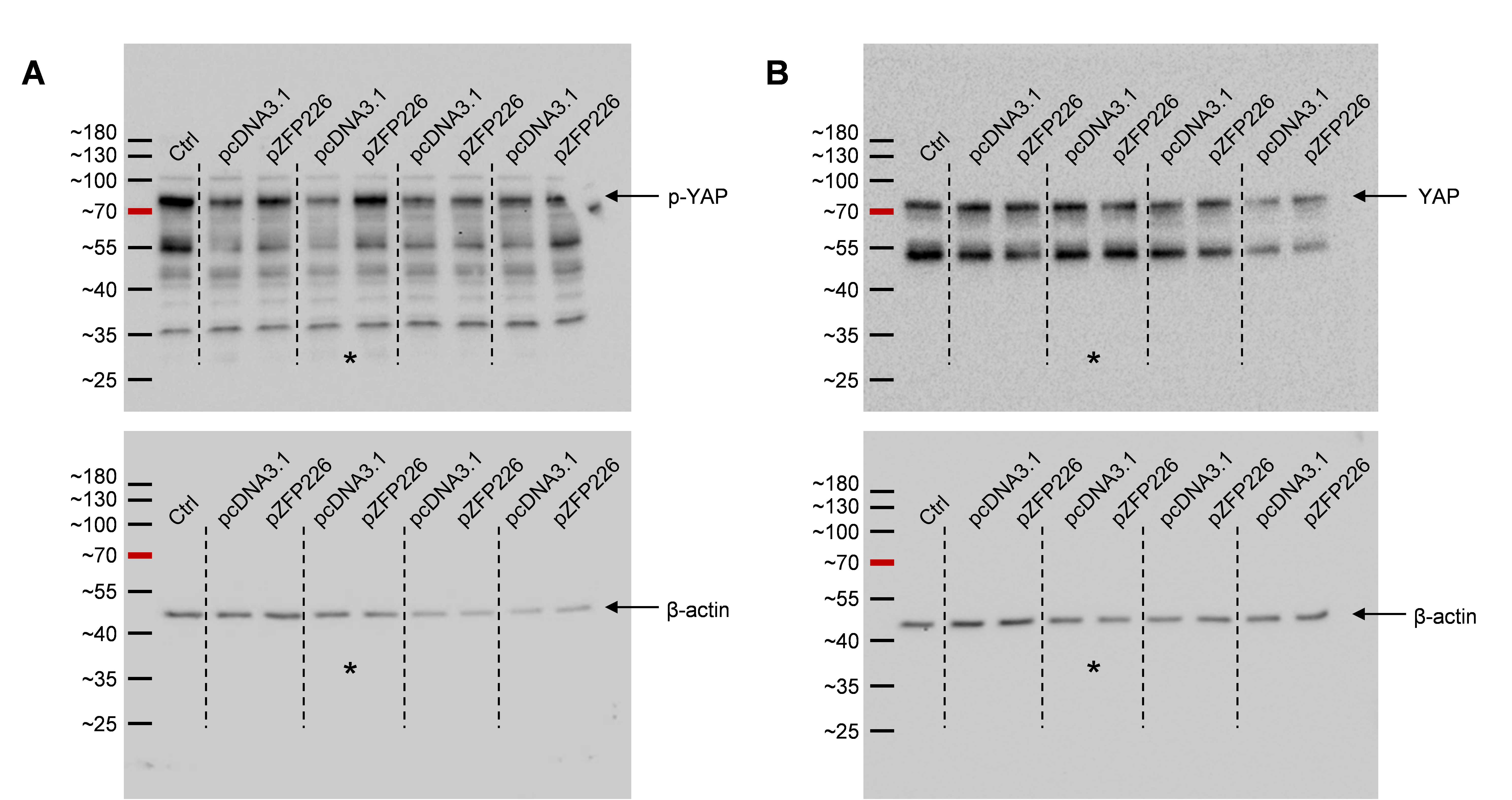
**

**ZFP226 overexpression enhances YAP phosphorylation.** Uncropped images of the blots used in Fig. 4A. ZFP226 vector was transfected into IHKE cells followed by western blot detection using **(A)** anti-pYAP (Ser127; Cell Signaling; 1:1000), **(B)** anti-YAP (Santa Cruz Biotechnology; 1:1000), and anti-rabbit secondary antibody (Santa Cruz Biotechnology; 1:20000). Sample loading was controlled by β-actin detection (Cell Signaling; 1:5000) and anti-rabbit secondary antibody (Santa Cruz Biotechnology; 1:10000). Asterisk marks the cropped image used in Fig. 4A. Ctrl = high YAP phosphorylation (cells were starved for 1 h using DMEM without fetal bovine serum).

**Supplementary figure S6**


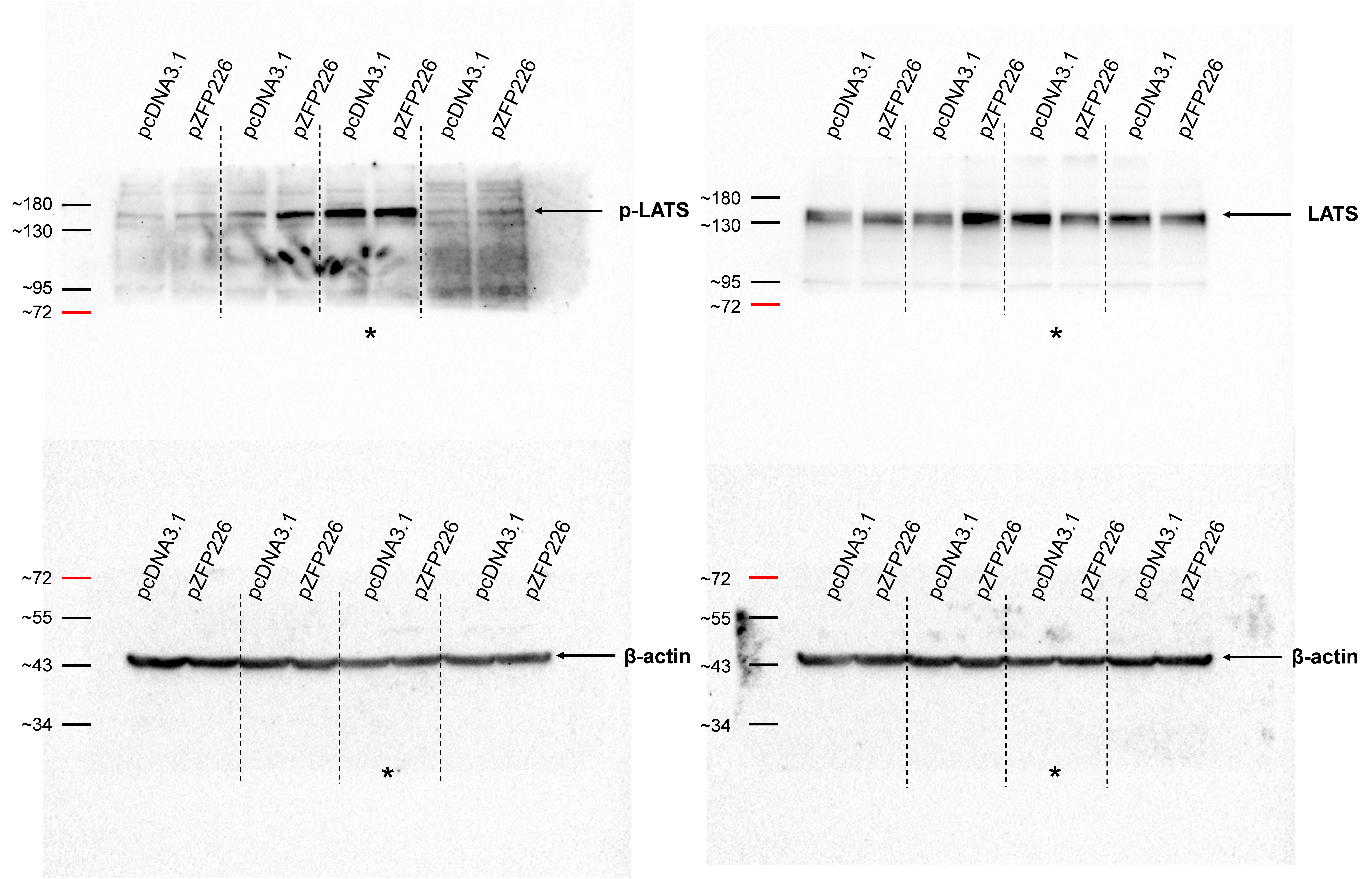


**ZFP226 overexpression enhances LATS1 phosphorylation.** Uncropped images of the blots used in Fig. 4B. ZFP226 vector was transfected into IHKE cells followed by western blot detection using **(A)** anti-pLATS1 (Thr1079; Cell Signaling; 1:500), **(B)** anti-LATS (Merck; 1:1000), and anti-rabbit secondary antibody (Merck; 1:20000). Sample loading was controlled by β-actin detection (Cell Signaling; 1:10000) and anti-rabbit secondary antibody (Merck; 1:20000). Asterisk marks the cropped image used in Fig. 4B.
